# Tuning the anticancer activity of a novel pro-apoptotic peptide using gold nanoparticle platforms

**DOI:** 10.1038/srep31030

**Published:** 2016-08-04

**Authors:** Mohammad Akrami, Saeed Balalaie, Saman Hosseinkhani, Mohsen Alipour, Fahimeh Salehi, Abbas Bahador, Ismaeil Haririan

**Affiliations:** 1 Department of Pharmaceutical Biomaterials and Medical Biomaterials Research Center, Faculty of Pharmacy, Tehran University of Medical Sciences, Tehran, Iran; 2 Peptide Chemistry Research Center, K. N. Toosi University of Technology, Tehran, Iran; 3 Department of Biochemistry, Faculty of Biological Sciences, Tarbiat Modares University, Tehran, Iran; 4 Department of Nanobiotechnology, Faculty of Biological Sciences, Tarbiat Modares University, Tehran, Iran; 5 Institute of Biochemistry and Biophysics, Department of Biochemistry, University of Tehran, Tehran, Iran; 6 Department of Microbiology, School of Medicine, Tehran University of Medical Sciences, Tehran, Iran; 7 Department of Pharmaceutics, Tehran University of Medical Sciences, Tehran, PO. Box: 14155-6451, Iran

## Abstract

Pro-apoptotic peptides induce intrinsic apoptosis pathway in cancer cells. However, poor cellular penetration of the peptides is often associated with limited therapeutic efficacy. In this report, a series of peptide-gold nanoparticle platforms were developed to evaluate the anticancer activity of a novel alpha-lipoic acid-peptide conjugate, LA-WKRAKLAK, with respect to size and shape of nanoparticles. Gold nanoparticles (AuNPs) were found to enhance cell internalization as well as anticancer activity of the peptide conjugates. The smaller nanospheres showed a higher cytotoxicity, morphological change and cellular uptake compared to larger nanospheres and nanorods, whereas nanorods showed more hemolytic activity compared to nanospheres. The findings suggested that the anticancer and biological effects of the peptides induced by intrinsic apoptotic pathway were tuned by peptide-functionalized gold nanoparticles (P-AuNPs) as a function of their size and shape.

In most types of cancer, genetic mutations lead to generation of a collection of drug-resistant cancer cells. This Population of mutated cells evade apoptosis, spread to distant organs and usually lead to death of most cancerous patients. Due to the low efficiency of most conventional methods, there is an urgent need to develop new therapeutic drugs^1^,^2^. Anticancer peptides (ACPs) that modulate specific molecular pathways in cancer cells are potential candidates for therapeutic purposes^2^. Previous studies have demonstrated that KLA peptides with a sequence of (KLAKLAK)_2_ can induce apoptosis in cancer cells^3^,^4^. This group of peptides facilitates the intrinsic apoptosis pathway via disrupting the mitochondrial outer membrane^5^,^6^. However, their impossibility to enter the cells has limited their therapeutic efficacy^6^. Sequence engineering of peptides and use of delivery systems as two well-known approaches are employed in order to improve ACP efficacy^7^,^8^. In the first approach, computational methods are used to design peptides with a higher anticancer activity^8^,^9^, while in delivery systems, various delivery vehicles, including cell penetration peptides (CPP), biopolymers and nanoparticles, have been developed to facilitate cellular uptake of pro-apoptotic peptides^5^,^9^,^10^,^11^.

Among the mentioned carriers, AuNPs offer a safe delivery platform and represent a collection of light responsive properties, which have been used for cancer nanomedicine^5^,^7^,^10^,^11^,^12^,^13^. However, a rational coupling strategy is considered as a prerequisite parameter for development of an ideal peptide-AuNP platform^5^,^14^,^15^. In this regard, disulfide linkers such as alpha-lipoic acid (LA) ensure a proper particle stability and remove the inherent cytotoxicity of Cetyl Triethyl Ammonium Bromide (CTAB) surfactant^15^,^16^,^17^,^18^,^19^,^20^,^21^,^22^,^23^,^24^.

Furthermore, size- and shape-dependent properties of AuNPs provide a unique advantage for design of tunable therapeutic platforms^25^. Indeed, these dimensional properties are able to determine the intracellular fate of nanoparticles^14^,^25^. Recently, it has been found that the conjugation with AuNP increases the anticancer efficacy of ACP^26^. However, the effect of AuNP architecture on the biological responses of KLA peptide has not yet been demonstrated. In the current study, a series of ACP-gold platforms were designed to evaluate the effect of AuNPs’ size and shape on the pro-apoptotic activity of ACPs. Briefly, a novel ACP with an amino acid sequence of WKRAKLAK, in conjugation with LA was synthesized and attached to gold nanospheres (AuNSs) and gold nanorods (AuNRs) with different sizes. The designed gold nanoparticle platforms efficiently delivered the ACP to cancer cells, disrupted the mitochondrial membrane and adjusted the pro-apoptotic activity regarding geometrical properties of nanoparticles.

## Results

### Synthesis and characterization of LA-Peptide and gold nanoparticles

**Figure 1:**
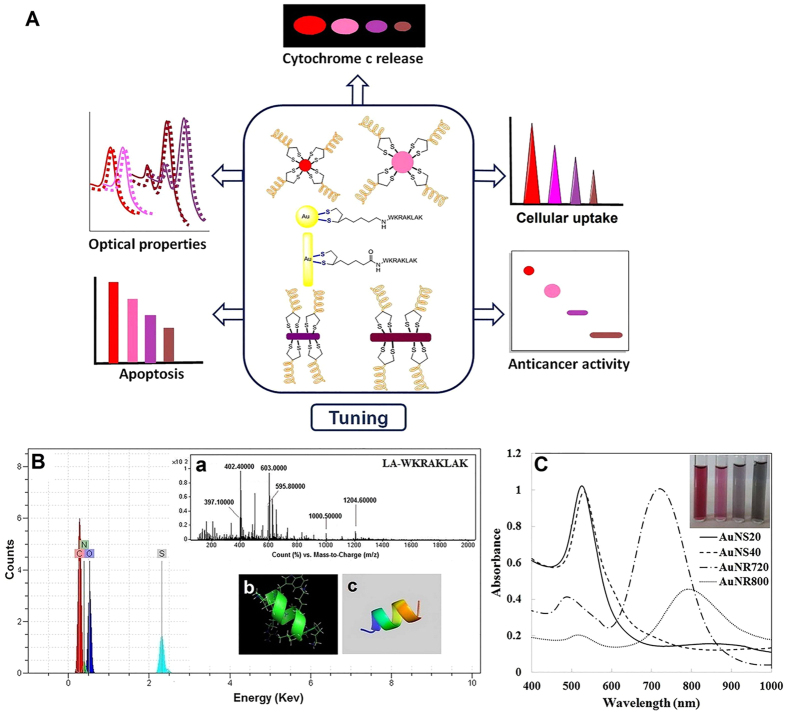
Characterization of LA-Peptide and gold nanoparticles. (**A**) Schematic representation of tuned biological responses of ACP by gold nanoparticle platforms; (**B**) Elemental analysis of the LA-Peptide sample by EDX. Inset (**a**) shows the mass spectrum of the purified LA-Peptide conjugate. Inset b and c are 3D structures of the peptide with and without side chain, respectively; (**C**) UV-Vis spectra of synthesized Au nanospheres and Au nanorods. The inset shows the image of prepared AuNS20, AuNS40, AuNR720 and AuNR800 from left to right.

A new sequence of anticancer peptides was designed by AntiCP prediction server based on support vector machine classifiers (SVM). KLA sequence was used as a template and AntiCP server proposed a series of potentially anticancer sequence with various SVM prediction scores (Table S1). The KRAKLAK sequence showed the highest score among the proposed sequences in terms of their anticancer property, positive charge and water solubility in the first stage^9^. After that, a Trp residue was improvised at the N-terminal position to provide a UV-active probe (Supplemental Figures S1 and S2)^27^. Analysis of the final sequence, WKRAKLAK, by AntiCP server indicated a good anticancer potency (Supplemental Table S1). Furthermore, the designed peptide was structurally predicted by I-TASSER server with a proper RMSD (−0.08). The results showed that alpha helix was the main secondary structure for this sequence in similar to its initial KLA peptide^28^ (Fig. 1B, inset b,c).

As shown in Figure S3A,B, the solid phase-synthesized LA-Peptide was successfully purified, with purity more than 94%, by RP-HPLC using C18 column (Supplemental Tables S2 and S3). The energy dispersive X-ray (EDX) analysis was used to confirm the successful attachment of LA to peptide via a qualitative determination of sulfur element. As shown in Fig. 1B, the presence of sulfur peak in addition to C, N and H elements could confirm the conjugation of LA to peptide. Mass spectrometry (+) ESI-MS analysis also showed that the molecular weight of LA-Peptide was 1186.6 Da, which was similar to the calculated molecular weight (Fig. 1B, inset a). The acquired molecular weight by mass spectrometry was confirmed via peaks of LA-WKRAKLAK, typically found to be 1204.6, 595.8 and 397.1, calculated for [m+solvent]^+^, [(m+2H)/2]^+^ and [(m+3H)/3]^+^, respectively.

AuNSs with diameters of about 20 nm (AuNS20) and 40 nm (AuNS40) as well as AuNRs with aspect ratios of 3.2 (AuNR720) and 4.2 (AuNR800) were synthesized according to the method developed by Murphy *et al*. with a size variation of 10%^29^. Spectra of seed solution for AuNPs is shown in Figure S4. UV–Vis spectroscopy of nanospheres showed surface plasmon resonance (SPR) peaks at 520 nm and 533 nm for AuNS20 and AuNS40, respectively. Furthermore, two characteristic SPR peaks were detected at the visible and near infrared spectra for anisotropic AuNR720 and AuNR800 (longitudinal plasmon peaks of 720 nm and 800 nm). In addition, the AuNS20 and AuNS40 solutions showed red and pink colors, but AuNR720 nm and AuNR800 nm solutions displayed dark purple and brown colors, respectively (Fig. 1C).

### Functionalization and characterization

In the modification approach, thiol-gold bond is used to form self-assembled monolayers of biomolecules on gold surfaces^15^,^30^,^31^,^32^. However, it is reported that mono-dendate thiol-gold bond as dative linkage is weaker than covalent linkage^15^,^33^. Therefore, in this study, LA was selected as a bi-dendate thiol ligand in order to provide greater adhesion of peptides to NP surfaces and to increase the overall stability of peptide-AuNP conjugates. In this procedure, salt aging was carried out to decrease the repulsions among positive charges of remaining CTAB and ligands. The schematic representation of the LA-Peptide in conjugation with AuNP platform is shown in Fig. 1A.

**Figure 2:**
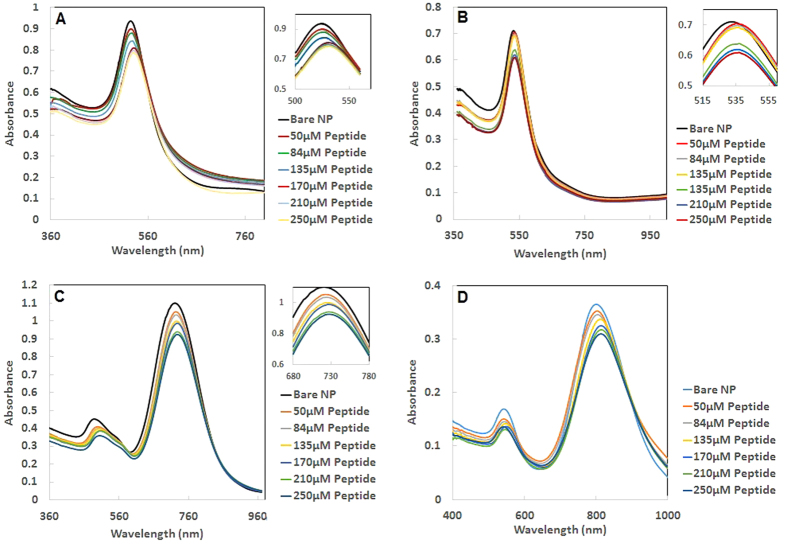
The change in plasmonic spectra of (**A**) AuNS20, (**B**) AuNS40, (**C**) AuNR720 and (**D**) AuNR800nm in the presence of different concentrations of the LA-Peptide conjugate (The insets clearly show the maximized spectra in the shifted wavelength range).

Next, functionalization of AuNPs with LA-Peptide was assessed using UV-Vis spectroscopy. Capping of AuNPs with different concentrations of LA-Peptide from 50 µM up to 250 µM resulted in plasmon peak red shift of around 2–7 nm, 1–4 nm, 2–6 nm and 3–13 nm for AuNS20, AuNS40, AuNR720 and AuNR800, respectively in comparison to bare nanoparticles. The shift was fixed at peptide concentration of 170 µM (Fig. 2, Table S4).

The observed red shift of SPR bond as a sign of changes in the dielectric properties of the medium, indicated the functionalization of AuNPs with the peptide (Fig. 2). Moreover, no change of color in the solutions, lack of strong shift and also absence of broadening in the SPR bond indicated the stability of functionalized AuNPs without significant particle aggregation.

**Figure 3:**
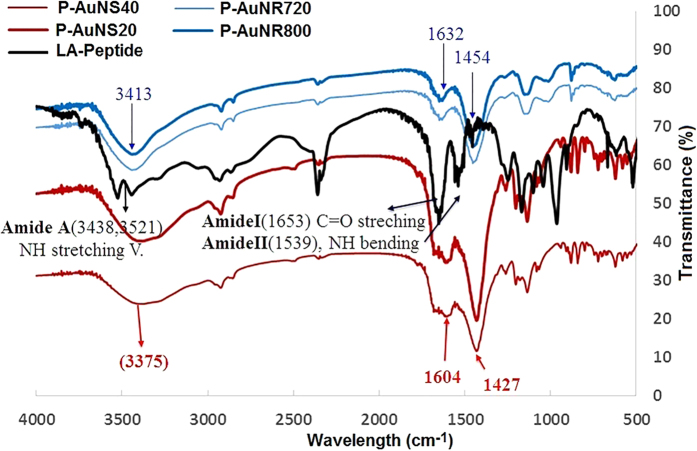
FTIR spectra of the LA-Peptide and the peptide functionalized AuNPs.

Capping of AuNPs with LA-Peptide was also investigated by FTIR measurements (Fig. 3). The presence of amide A, amide I, amid II and amide III bands in bioconjugated AuNPs corresponded to that in the free LA-Peptide, supported the existence of bonded peptide in the peptide-AuNPs. The amide A bond (3438 cm^−1^ and 3521 cm^−1^) in the free LA-Peptide shifted to broader peaks, at 3375 cm^−1^ and 3413 cm^−1^ in P-AuNSs and P-AuNRs, respectively, suggesting that a strong hydrogen binding was formed^34^,^35^. Furthermore, two peaks located at 1653 cm^−1^ and 1539 cm^−1^ in the amide I and amide II regions for free LA-Peptide were changed to 1604 cm^−1^ and 1427 cm^−1^ for P-AuNSs, respectively. Similarly, those bands shifted to 1632 cm^−1^ and 1454 cm^−1^ for P-AuNRs. The obtained data suggest that the hydrogen binding pattern and subsequently backbone conformation of LA-Peptide were affected by the LA-Peptide attachment to the gold surface.

Furthermore, it is generally reported that the appearance of a peak around 1655 cm^−1^ in a peptide can be assigned to α-helix conformation, and this supported structural prediction by I-TASSER server^36^ in the present study.

**Figure 4:**
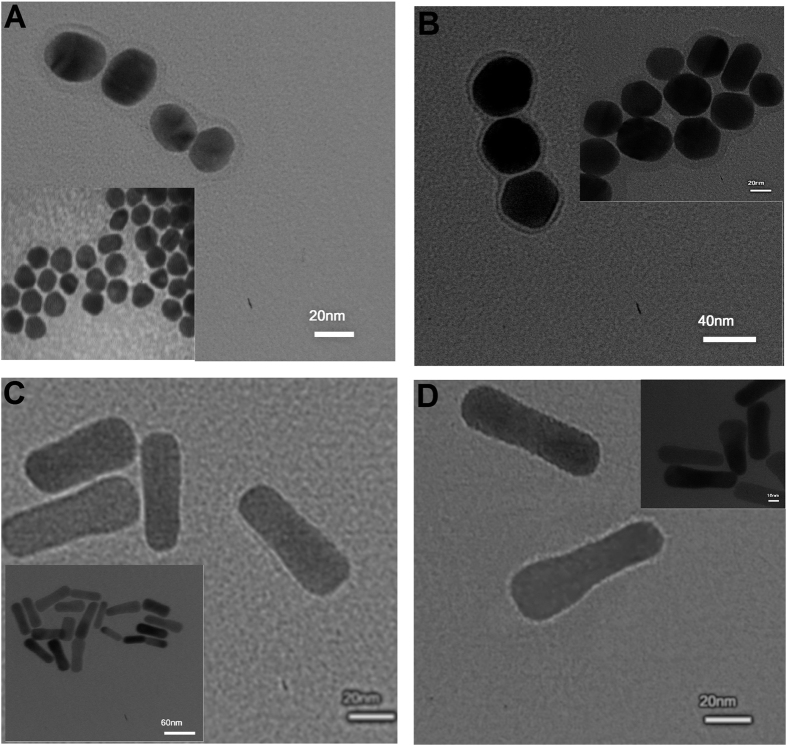
The zoomed HRTEM micrographs of the P-AuNSs with diameters of (**A**) ~20 nm and (**B**) ~40 nm; the P-AuNRs with (**C**) AR of ~3.2 and (**D**) AR of ~4.2. (The insets show the HRTEM images of the NPs with original sizes).

The size and morphology of peptide-AuNPs were examined by HRTEM (Table 1). The HRTEM results clearly showed that the LA-Peptide shell was conjugated on the AuNPs (Fig. 4A–D).

Two spherical shapes with mean diameters of 28.1 nm and 49.7 nm and average core sizes of 19.2 nm and 39.4 nm were obtained for P-AuNS20 and P-AuNS40, respectively. In addition, HRTEM images displayed mean longitudinal sizes of 58.0 nm and 66.7 nm for AuNR720 and AuNR800 both, including about 1–2 nm LA-Peptide shells.

Dynamic light scattering (DLS) analysis, on the other hand, revealed rises of about 5.9 nm and 10.78 nm in diameters for P-AuNS20 and P-AuNS40, respectively, with acceptable polydispersity index (PDI) due to functionalization by LA-Peptide (Table 1).

It is worthwhile to point out that the smaller sizes of AuNPs estimated by HRTEM than DLS could be attributed to hydration layer formation during measurement by DLS.

Furthermore, zeta potential value for bare AuNPs was higher than +20, which contributed to their CTAB coating. This value declined to about +6 when AuNPs were conjugated with LA-Peptide (Table 1). The as-prepared core shell of AuNPs was highly water dispersible.

To determine the amount of gold in unknown samples by atomic absorption spectroscopy (AAS), a standard calibration curve was prepared as shown in Figure S5. The number of AuNPs in samples was estimated from the average diameter of NPs based on TEM image, followed by geometrical calculations of the NP mass. The number of peptides was determined indirectly according to the standard calibration curve of the peptide (Supplemental Fig. S6A,B). The number of peptide/particle in P-AUNS20, P-AUNS40, P-AuNR720 and P-AuNR800 was estimated to be about 7598 ± 134, 48327 ± 345, 12824 ± 572 and 15103 ± 803, respectively.

### Cytotoxicity

The cytotoxic effects of bare AuNPs and peptide functionalized AuNPs as well as free LA-Peptides against both MCF-7 and metastaticT47D breast cancer cells were evaluated using the MTT assay. No significant toxicity was seen for the bare AuNSs and AuNRs. IC_50_ values of P-AuNR800, P-AuNR720, P-AuNS40 and P-AuNS20 for MCF-7 cells were 41.9, 21.25, 13.13 and 8.75 μg/mL, respectively, while for T47D cells they were 27.5, 9.25, 4.99 and 3.33 μg/mL, respectively. IC_50_ values in terms of LA-Peptide content were also calculated (Supplemental Table S5). The cell killing of MCF-7 cells upon peptide concentration has increased about 141, 128, 85 and 49 times for P-AuNS20, P-AuNS40, P-AuNR720 and P-AuNR800 compared to free LA-Peptide, respectively. Meanwhile, the more cell killing of T47D compared to MCF-7 was found for nanoparticles. However, the LA-Peptide solely showed cytotoxic effects at higher concentrations compared to P-AuNPs in both MCF-7 and T47D cell lines (Supplemental Figure S7A,B). The results demonstrated that spherically shaped nanoparticles suppressed cancer cell growth more efficiently than rod-shaped ones. Likewise, smaller AuNPs resulted in greater inhibition of cell growth. The data showed that cell viability in the presence of P-AuNPs was cell-type sensitive, so less cell inhibition by P-AuNPs occurred for MCF-7 in comparison to T47D cells.

**Figure 5:**
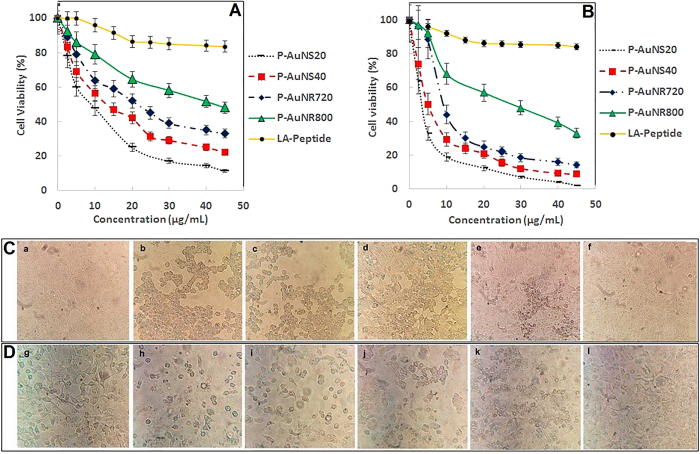
Cell viability assay of (**A**) MCF-7 and (**B**) T47D breast cancer cells exposed to AuNPs, P-AuNPs and the free LA-Peptide. (**C**) Representative photographs of morphological changes for MCF-7 (upside row) and (**D**) T47D breast cancer cells (down side row) in presence of PBS as control (a,g); P-AuNS20 (b,h), P-AuNS40 (c,i), P-AuNR720 (d,j), P-AuNR800 (e,k) and the free LA-Peptide (f,l) under the inverted microscope.

The differences in morphology of breast cancer cell lines in the presence of AuNPs at the concentrations equal to IC_50_ values were assessed using the inverted microscope (Fig. 5C,D). However, after treatment with nanospheres, cell rounding, detachment and sometimes shrinkage was clearly observed. The change in morphology was also observed with nanorod treatment but to a lesser extent than in the case of nanospheres. These differences all supported the cytotoxicity results.

### Hemolysis

Due to lack of hemocompatibility, when some pharmaceutical biomaterials come into contact with erythrocytes, hemoglobin may be released into the plasma. Therefore, the hemocompatibility test is considered very important before any clinical study^37^.

**Figure 6:**
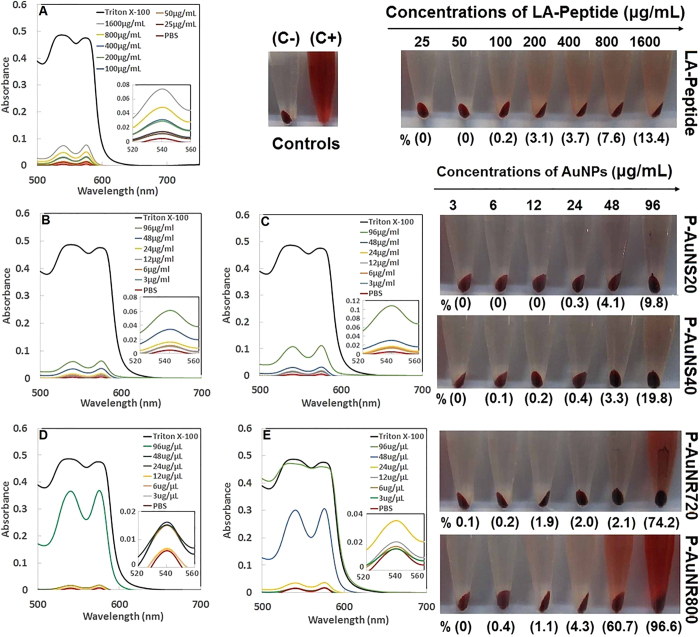
The hemolysis assay of RBSs exposed to (**A**) Free LA-Peptide, (**B**) P-AuNs20, (**C**) P-AuNS40, (**D**) P-AuNR720 and (**E**) P-AuNR800. The photographs of each treated sample as well as positive and negative controls (C+,C−) are represented on the right side of the curves. Numbers above the arrows show the serially-diluted concentrations of the LA-Peptide and P-AuNPs, while numbers in brackets under each photograph indicate the hemolytic percentages of related concentrations.

Hemolytic activity of AuNSs and AuNRs was tested using serially-diluted samples with concentrations from 3 to 96 μg/mL. As shown in Fig. 6, the color of supernatant was used as an indication of apparent hemolytic activity. Triton X-100, as positive control, displayed obvious hemolysis while no hemolytic activity was observed in the supernatant of PBS buffer, used as negative control. Greater hemolytic activity occurred for P-AuNR800 compared to P-AuNR720 (max. 99.6% vs. 74.2% at concentration of 96 μg/mL) and a similar result occurred for P-AuNS40nm in comparison to P-AuNS20 nm at the same concentration (max. 19.8% vs. 9.8%). Therefore, the highest amount of hemolytic activity was observed for P-AuNR800 treatment. According to the standard acceptance value (less than 5%), the blood compatibility of tested nanomaterials was confirmed for P-AuNR720 and P-AuNR800 at concentrations less than 48.0 and 24 μg/mL (Fig. 6D,E)^38^. The hemocompatible concentration less than 48 μg/mL was found for both P-AuNS40 and P-AuNR20 (Fig. 6B,C). However, for free LA-Peptide, this concentration was increased to 400 μg/mL, with 3.7% hemolysis (Fig. 6A).

### Cellular Uptake

**Figure 7:**
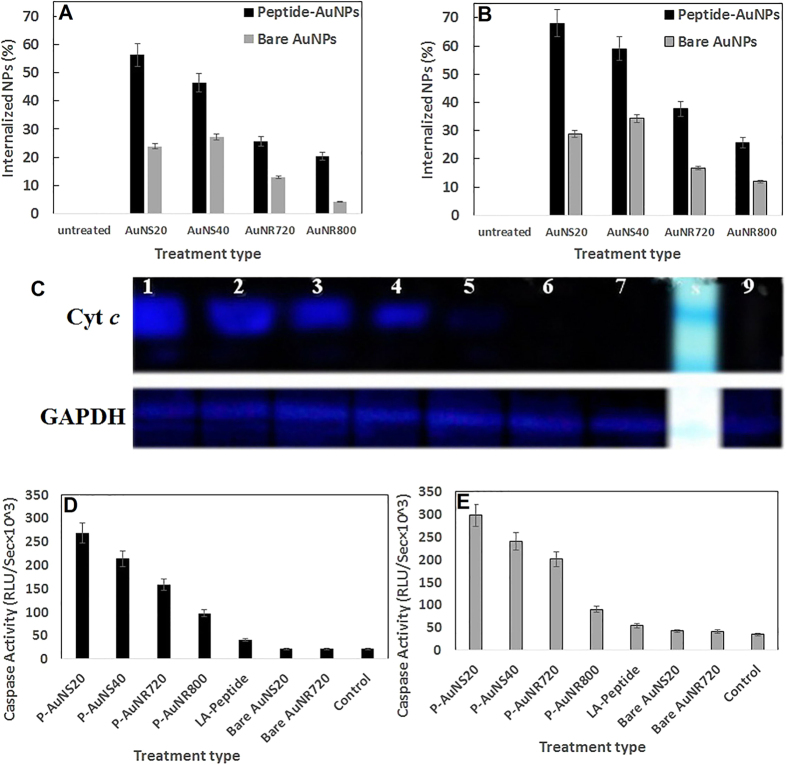
Cellular uptake of the bare and peptide-functionalized gold nanospheres and nanorods by (**A**) MCF-7 and (**B**) T47D breast cancer cells within 24 h of quantification using atomic absorption spectroscopy. Immunoblotting of the released cytochrome c from MCF-7 cell lines (**C**) after induction by (1) P-AuNS20, (2) P-AuNS40, (3) P-AuNR720, (4) P-AuNR800 and (5) the free LA-Peptide, (6) bare AuNS20 and (7) bare AuNR720. Lans of 8 and 9 demonstrate Ladder and untreated cells. Caspase-3/7 activity of (**D**) MCF-7 and (**E**) T47D cell lines after apoptotic induction by P-AuNS20, P-AuNS40, P-AuNR720, P-AuNR800 and the LA-Peptide. Bare nanospheres, nanorods and also PBS were used as controls. The data are represented in terms of means ± S.D of triplicate experiments.

Cell internalization of AuNPs was investigated and quantified by AAS with respect to their size and shape. For both cell lines, the cellular uptake percentage increased upon treatment with P-AuNPs compared to the related bare AuNPs (Fig. 7A,B). In addition, the most efficient cellular uptake of P-AuNPs and bare AuNPs occurred in the presence of P-AuNS20 nm and bare AuNS40 nm. This means that the uptake pattern of bare nanospheres was altered by peptide functionalization.

On the other hand, less uptake was observed for larger bare and functionalized nanorods. Furthermore, greater uptake by T47D cells was shown in comparison to that by MCF-7 cells.

### Cytochrome *c* release

Release of cytochrome *c* from mitochondria in response to apoptogens is an important step of apoptosis in the intrinsic pathway. It is known that introduced pro-apoptotic peptides can trigger the mitochondria-dependent apoptosis in which cytochrome *c* efflux into the cytosol occurs after permeabilization and swelling of the mitochondria membrane^4^.

Effect of NPs on cytochrome *c* release was investigated with anti-cytochrome *c* using western blotting after 24 h of treatment of MCF-7 cells with AuNPs. As shown in Fig. 7C (Lan 1–5), cytochrome *c* release was significantly observed for P-AuNS20, P-AuNS40, P-AuNR720, P-AuNR800 and partially observed for the free peptide. Furthermore, no cytochrome *c* was observed upon treatment with bare AuNS20 and bare AuNR800 (Lan 6 and 7) as well as the untreated sample (Lan 9).

### Caspase 3/7 activity

Caspase 3/7 as the main executioner caspases in the intrinsic apoptotic pathway could be detected if their inactive pro-form is cleaved by upstream caspases after cytochrome *c* release and apoptosome formation^39^. Caspase 3/7 activation capability was evaluated after treatment of cancer cell lines with P-AuNPs (Fig. 7 D,E). After incubation of MCF-7 cells with P-AuNS20, P-AuNS40, P-AuNR720, P-AuNR800 and free LA-Peptide for 24 h, the activity of caspase-3/7 increased by nearly 12.8, 10.2, 7.5, 4.6 and 1.6 times respectively compared to the untreated cells (negative control).

However, for the treated T47D cells with the same nanoparticles, increases of 8.7, 7.0, 5.9, 2.6 and 1.6 times were obtained. On the other hand, up to 24 h no significant difference in caspase3/7 activity was found among the cells treated with bare AuNS20, bare AuNR720 and PBS (as controls).

## Discussion

Mitochondrial targeting has been considered as a novel approach for treatment of different types of cancers^4^,^26^,^39^,^40^. This strategy not only restores efficient cell death pathway in cancer cells, but also it perturbs signaling transduction and mitochondrial respiration of these cells, the latter resulting in high consumption of energy supplies^39^,^41^.

Recently, peptides that induce cell death via apoptosis in cancer cells have attracted ever-increasing attention^2^,^42^. For instance, cationic AMPs with pro-apoptotic effects have shown a strong antitumor activity^43^.

However, cell internalization is perceived as a major obstacle for development of pharmaceutically useful peptides such as bivalent KLA peptides with anticancer activity^44^,^45^.

In this study, a water soluble and cationic peptide, WKRAKLAK, was designed via AntiCP server. However, toxicity assessment of the synthesized peptide demonstrated a nearly high IC_50_ (Supplemental Figure S7). It is speculated that the LA-Peptide itself had weak cell penetration capability, which was consistent with the properties of (KLAKLAK)_2_ peptide^6^. Recently, anticancer activity of (KLAKLAK)_2_ peptide has strongly increased by nanoassembly of the peptide on AuNPs^26^. Therefore, for the present study, it was hypothesized that the peptide efficacy and delivery would be improved via peptide attachment on the surface of AuNPs.

As expected, AuNPs demonstrated enhanced peptide anticancer efficacy, which was completely dependent upon the size and shape of AuNPs. In addition, P-AuNS20 induced a higher cytotoxicity compared to P-AuNS40 and P-AuNRs (Fig. 5).

On the basis of toxicity results, the mechanisms that govern the size- and shape-dependent behavior of P-AuNP platforms were also investigated. As shown in Fig. 7A, the results of atomic absorption revealed higher uptake for smaller nanospheres compared to other sizes and shapes of P-AuNPs, which is in agreement with the literature^46^,^47^. The observed difference among nanoparticles could be attributed to different radii of curvature as well as surface area/volume ratios and thereupon different peptide density on each NP surface^14^,^48^. Moreover, the favorite configuration of the peptide can be another key point that determines the uptake rate of nanoparticles^49^.

On the other hand, P-AuNR800 showed the least uptake among other functionalized nanoparticles. The result may be due to occupation of more cellular receptors by long axis of nanorods with high AR compared to other sizes and shapes^47^.

It has also been reported that different patterns of adsorbed plasma protein for various sizes/shapes influence the cellular uptake^50^. More studies will be required to understand the mechanism involved in cellular uptake of LA-WKRAKLAK functionalized AuNPs.

In another attempt, to clarify the mechanism behind the higher anticancer activity of P-AuNP platforms, the intrinsic pathway of apoptosis was evaluated. As shown in Fig. 7C, although the designed WKRAKLAK peptide exhibited a partial cytochrome *c*-mediated pro-apoptotic activity, no pro-apoptotic activity was seen for the bare nanoparticles.

Conjugation of this novel ACP to AuNPs significantly boosted mitochondrial disruption and pro-apoptotic activity in a dimensionally-dependent manner (Fig. 7). The results suggested that AuNPs provide multivalent ACP on their surface and subsequently intensify their pro-apoptotic activity.

It was also observed that P-AuNPs not only increased the anticancer activity but also showed a hemocompatible behavior (Fig. 6), which is an important factor for *in vivo* administration of anticancer therapeutic platforms^37^.

In conclusion, AuNP provided a safe and hemocompatible delivery platform to improve the anticancer efficacy of ACP. In addition, by adjusting the size/shape of AuNPs, not only it was possible to tune their optical properties but also their biological responses were tuned. Furthermore, the designed P-AuNPs in this study targeted the mitochondrial membrane due to caspase 3/7 activation following the cytochrome *c* release. The findings suggest that these tunable mitochondrial targeting platforms are appropriate for utilization in cancer therapeutics.

## Conclusion

Small peptides with pro-apoptotic activity can be regarded as promising anticancer agents if a delivery system improves their efficacy. Here, ACP-functionalized gold platforms were developed to enhance the anticancer potency of a novel pro-apoptotic peptide. The size- and shape-dependent nature of these platforms provided a suitable tool for regulating the pro-apoptotic activity of designed ACPs. These ACP-gold platforms could induce apoptosis in drug resistant cancer cell lines. Our finding suggest that ACP conjugated with small-sized gold nanosphere might be a promising therapeutic for cancer treatment.

## Methods

### Materials

Gold tetrachloroaurate (HAuCl_4_.3H2O), Sodium borohydride (NaBH_4_), Silver Nitrate, CTAB (Cetyl triethyl ammonium bromide), Ascorbic acid and NaCl were purchased from Sigma Company. Alpha lipoic acid was obtained from ACROS organics.

UV-Vis absorbance spectra was collected by a UV-Vis-NIR spectrophotometer (JASCO, V-530). Deionized water was used for all preparations. Prior to the experiments, Aqua regia (volume of HCl: HNO_3_ in a ratio 3:1) was used to clean glass wares. The glass wares were rinsed with deionized water before usage.

### Preparation of Au seeds

Gold seed solution (10 mL) was prepared as published^51^ (see supporting Information).

### Synthesis of CTAB-capped gold nanospheres (20 nm, 40 nm)

The Au nanosphere shapes were produced by acrorbic acid mediated reduction method. For synthesis of AuNSs with a diameter of about 20 nm (AuNS20), growth solutions of AuNS20 (7.2 mL of 0.1 M CTAB and 0.225 mL of 0.01M HAuCl_4_·3H_2_O) and AuNS40 (6.4 mL of 0.1 M CTAB and 0.200 mL of 0.01 M HAuCl_4_·3H_2_O) were separately mixed with solution of fresh ascorbic acid (0.050 mL, 0.1 M). After that, 0.1 mL and 0.026 mL of seed solution were transferred to the stirring mixtures, respectively. Finally, these solutions were stirred strongly for 10 sec until a wine-red solution was obtained. The mixtures were left undisturbed overnight.

### Synthesis of CTAB-capped gold nanorods

For preparation of AuNRs with longitudinal peak (LP) located at about 720 nm (AuNR720), AgNO_3_ (0.01 M, 0.015 mL) was added to the mixture of growth solution (5 mL of 0.1 M CTAB and 0.125 mL of 0.01 M HAuCl_4_·3H_2_O) under stirring. This was followed by addition of freshly prepared ascorbic acid (0.025 mL, 0.1 M). Finally, seed solution (0.015 mL) was transferred to the mixed solution and after 10 sec of stirring, it was incubated overnight. The same procedure was used for preparation of AuNR with LP located at 800 nm (AuNR800). Briefly, the nanorod solution was produced through mixture of CTAB (5 ml), HAuCl_4_·3H_2_O (0.250 mL), AgNO_3_ (0.050 mL), ascorbic acid (0.028 mL) and seed solutions (0.006 mL) with the same concentrations.

### Peptide design, synthesis, conjugation with LA and characterization

AntiCP web server was used to predict the anticancer efficacy of the given sequence so that it could meet the purposes of the present study^52^. The peptide synthesis and its conjugation with LA are described in supplementary data^53^,^54^. After purification of peptide conjugate by semi-preparative RP-HPLC, the purity of the lyophilized peptide was examined by analytical high-performance liquid chromatography (HPLC, Purity >95%). To confirm the conjugation of LA to the peptide, elemental compositions of LA-Peptide was also evaluated by EDX analysis. The molecular weights were confirmed by mass spectrometry (+) ESI-MS. The tertiary structure of functional peptide was modeled by I-TASSER server^55^. The secondary structure of peptides were assigned using DSSP. PyMol iZ3D SOFWARE was used to visualize the 3D structure of peptide.

### Functionalization of gold nanoparticles with peptide

Before functionalization, excess CTAB of AuNS and AuNR solutions were removed by centrifuging twice at 18000 rpm for 20 min and at 9000 rpm for 10 min, respectively. For better ligand exchange, the precipitated nanoparticles were redispersed in NaCl solution (10 mM), then 10 μl of peptide (10 μg/mL) was added to dispersions, mixed and incubated for 72 h at room temperature. Peptide-functionalized AuNS and AuNR (P-AuNS and P-AuNR) were precipitated by centrifuging at 9000 rpm and 6000 rpm for 30 min, respectively. This was followed by three times of washing with water. Finally, nanoparticles were redispersed in water for future use. In addition, the functionalization process of AuNPs was performed in the presence of different concentrations of peptide.

### Physicochemical properties of peptide functionalized gold nanoparticles

The functionalized AuNPs were characterized using various analytical techniques. To evaluate the optical properties of gold nanoparticle solutions, their UV-Vis spectra were recorded on a JASCO spectrometer (UV-1700) using quartz cuvettes. Morphology and sizes of the NPs were characterized using HRTEM (Philips, CM30) operating at 200 kV potential. In all cases, an amount of 3 μL of the diluted colloidal dispersions was drop-cast carefully onto a carbon-supported copper grid and dried in air before imaging. The hydrodynamic size and zeta potential of the particles were determined by Malvern Zetasizer Nano ZS90 at room temperature.

For FTIR analysis, the samples of gold colloids were lyophilized; then the dried particles were mixed with KBr and pressed as pellet. The infrared spectra of samples were recorded on a schimatzu spectrophotometer with IR solution software at frequencies ranging from 400 to 4000 cm^−1^ with 25 scans. The gold nanoparticle concentrations were obtained by AAS. An independent method was used for determination of the number of peptides per particle. LA-Peptide solutions (10 μM) were added to gold nanoparticle suspensions. After 72 h of incubation, the AuNPs were centrifuged to remove the nanoparticles. Absorbance of the supernatant at 280 nm was measured to determine the concentration and number of remaining free peptides in supernatant.

### Cell viability assay (MTT)

MTT colorimetric assay was used to metabolically quantify viable breast cancer cells after exposure to AuNPs. In brief, MCF-7 and T47D cells (Pasteur Institute, National Cell Bank of Iran) were seeded into 96-well plates at a density of about 20000 cells per well in RPMI 1640 medium (200 μL). Medium was supplemented with L-glutamine (2.0 mM), penicillin (50 U/mL), streptomycin (0.05 mg/mL) and 10% heat inactivated FBS in the presence of 5% CO_2_ at 37 °C. After 24 h, the medium was replaced with 200 μL fresh media containing AuNPs (10 μL) at different concentrations (2.5, 5.0, 10.0, 15.0, 20.0, 25.0, 30.0 and 45.0 μg/mL). Control cells were incubated with a fresh culture medium. Following a further incubation for 48 h, the MTT reagent (final concentration being 0.5 mg/mL) was added to each well and incubated for 4 h to detect the metabolically active cells. Finally, the incubation solution was then removed and the formazan crystals were solubilized by addition of DMSO. The absorbance of each well was measured at 570 nm by a microplate reader (Stat Fax-2100, AWARENESS, Palm City, USA). The percentage of cell viability was calculated using the following formula:
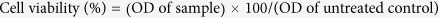


The results were obtained from triplicate determinations. Morphologies of the cell lines exposed to NPs were compared by phase contrast microscopy.

### Hemolysis

Hemolysis assay was performed against AuNPs according to previously published procedure^56^. The absorbance values of supernatants at 540 nm were determined using Jasco-530 spectrophotometer. Finally, the hemolysis percentage of the different samples was calculated^56^.

### Determination of cellular uptake of AuNPs

To determine the cellular uptake of P-AuNRs, P-AuNSs and related bare nanoparticles by breast cancer cells, T47D and MCF-7 cell lines were plated in 24-well culture plates at 1 × 10^6^ cells per well and allowed to achieve 85% confluency. The cells were incubated in the presence of AuNPs for 24 h. After that, the cells were washed 3 times with PBS buffer to remove the unbound P-AuNPs. The cells were then trypsinized and the pellet was dissolved with 1 mL of aqua regia (HCl:HNO3, 3:1). Cellular uptake percentage as the gold content of the samples was quantified by AAS technique (Varian AA240FS, USA) as mentioned above.

### Cell extract preparation

Cell lysates was obtained by cytosolic fractionation method^57^ (see supporting Information).

### Western blotting

Cytochrome *c* release from mitochondria of MCF-7 breast cancer cells was evaluated using western blot immunoassay^57^. Briefly, the same amounts of total protein in cytosolic fractionation were loaded into the wells of SDS-PAGE. The separated proteins on a 15% gel were transferred onto a nitrocellulose membrane by transfer buffer (25 mM Tris base, 190 mM glycine, 20% methanol, pH adjusted to 8.3). The membrane was blocked with TBST buffer containing 5% (w/v) BSA at room temperature. After washing, the membrane was incubated with Anti-cytochrome c and Anti-GAPDH primary antibodies. The corresponding horseradish peroxidase coupled secondary antibody was then incubated with membrane for 1 h at RT. The membrane was developed with chemiluminescence reagent Lumigen TMA-6 (GE Healthcare UK limited, Buckinghumshire, UK) and images were captured using western blot imaging system (Sabz Biomedicals, Iran).

### Apoptosis

The apoptosis assessments in all breast cancer cell lines were carried out using the Caspase-Glo 3/7 luminescent assay system (Promega). The cell lysates were prepared by cytosolic fractionation as described above. In brief, Caspase-Glo^®^ 3/7 Reagent (5 μL) was added to equal total protein concentrations of control cells (no treatment) and treated cells (5 μL), followed by gentle mixing for 30 seconds. Incubation was followed for 30 min at room temperature. The luminescence of each sample was measured by a luminometer (Berthold Detection System, Germany).

### Statistical Analysis

Experimental data were processed using Microsoft Excel 2013 software in which the results were presented as means ± standard deviation of three independent experiments. Significant differences between means were conducted using a t-test, with statistical significance when P value < 0.05.

## Additional Information

**How to cite this article**: Akrami, M. *et al*. Tuning the anticancer activity of a novel pro-apoptotic peptide using gold nanoparticle platforms. *Sci. Rep.* **6**, 31030; doi: 10.1038/srep31030 (2016).

## Supplementary Material

Supplementary Information

## Figures and Tables

**Table 1:** Hydrodynamic diameters, HRTEM sizes, aspect ratio and zeta potentials of prepared nanoparticles.

Nick name	HRTEM Measurement		Average size measured by DLS (nm)	PDI	Zeta potential (mV)
	Average size (nm)	Aspect ratio			
P-AuNS20 core	19.2 ± 2.9	—	24.31 ± 0.71	0.279	31.14
P-AuNS20	28.1 ± 3.3	—	30.21 ± 0.85	0.254	6.65
P-AuNS40 core	39.4 ± 2.4	—	44.31 ± 2.6	0.234	22.63
P-AuNS40	49.7 ± 2.6	—	55.09 ± 4.3	0.302	6.06
P-AuNR720 core	—	3.2 ± 0.5	—	0.293	25.80
P-AuNR720	—	2.9 ± 0.5	—	0.310	4.90
AuNR800 core	—	4.2 ± 0.3	—	0.342	24.30
P-AuNR800	—	3.5 ± 0.4	—	0.363	5.20
