# Supplementary material for: Tuning the anticancer activity of a novel pro-apoptotic peptide using gold nanoparticle platforms
Source: Sci Rep. 2016 Aug 4;6:31030. doi: 10.1038/srep31030 (PMC4977985; doi:10.1038/srep31030)
Supplement: Supplementary Information [file srep31030-s1.pdf]

Supporting Information

Tuning the anticancer activity of a novel pro-apoptotic peptide using gold nanoparticle platforms

Mohammad Akrami[1], Saeed Balalaie[2], Saman Hosseinkhani[3,4], Mohsen Alipour[4], Fahimeh Salehi[5], Abbas Bahador[6] & Ismaeil Haririan[1,7,*]
[1]Department of Pharmaceutical Biomaterials, and Medical Biomaterials Research Center, Faculty of Pharmacy, Tehran University of Medical Sciences, Tehran, Iran
[2]Peptide Chemistry Research Center, K. N. Toosi University of Technology, Tehran, Iran,
[3]Department of Biochemistry, Faculty of Biological Sciences, Tarbiat Modares University, Tehran, Iran.
[4]Department of Nanobiotechnology, Faculty of Biological Sciences, Tarbiat Modares University, Tehran, Iran.
[5]Institute of Biochemistry and Biophysics, Department of Biochemistry, University of Tehran, Tehran, Iran
[6]Department of Microbiology, School of Medicine, Tehran University of Medical Sciences, Tehran, Iran
[7]Department of Pharmaceutics, Tehran University of Medical Sciences, Tehran, PO. Box: 14155-6451, Iran

*haririan@tums.ac.ir

Preparation of Au seeds. Briefly, 0.250 mL of an aqueous $HAuCl_4 \cdot 3H_2O$ (0.01M) was added to 9.75 mL of a CTAB solution (0.10 M) in a glass tube and mixed by inversion. After that, 0.700 mL freshly prepared solution of ice-cold $NaBH_4$ (0.01M) was added to the mixture, followed by rapid mixing for 10 min. The resultant pale brown-yellow seed solution from yellowish solution was kept undisturbed at room temperature for 2-5 hours to hydrolyze the unreacted $NaBH_4$. The produced nanoparticles (with size <4 nm) as gold seeds were used to make AuNS and AuNR solutions.

Peptide design and synthesis. WKRAKLAK was synthesized by standard Fmoc solid phase protocols on a trityl Chloride resin. Briefly, after swelling the clear resin in DCM, first amino acid (dissolved in DMF:DCM in advance) and DIPEA were added to a reaction vessel. Amino acid loaded resin was then capped with a DCM:DIPEA:MeOH solution. Fmoc deprotection was achieved by short treatment of resin with 25% pipyridine in DMF. Coupling reaction of the next amino acids was performed in presence of TBTU and DIPEA, dissolved in a mixture of DMF:DCM. Monitoring of each coupling reaction was performed using Kaiser calorimetric test. The coupling and Fmoc deprotection steps were repeated until all of the amino acids were coupled onto the resin.

Conjugation of peptide with alpha-lipoic acid (LA) and characterization. After coupling the last amino acid, LA (-COOH) was coupled to ($-NH_2$) sequence in presence of quantitative amounts of TBTU and DIPEA. 1% TFA in DCM was used for cleavage of the LA-Peptide from the resin. The filtered solution was treated with 4% methanolic pyridine immediately. After evaporation and precipitation, side-chain protecting groups were removed from the

obtained conjugate by a cleavage cocktail (trifluoroacetic acid: triisopropyl silane: methanol) under gentle stirring for 2h. The cleavage cocktail was concentrated to 1.0 mL by a rotary evaporator under reduced pressure, precipitated in cold anhydrous diethylether and dried in vacuum. The protecting groups were removed from the obtained conjugate by cleavage cocktail (trifluoroacetic acid: triisopropyl silane: methanol) under gentle stirring for 2h. The cleavage cocktail was concentrated to 1.0 mL by a rotary evaporator under reduced pressure, precipitated in cold anhydrous diethylether and dried in vacuum.

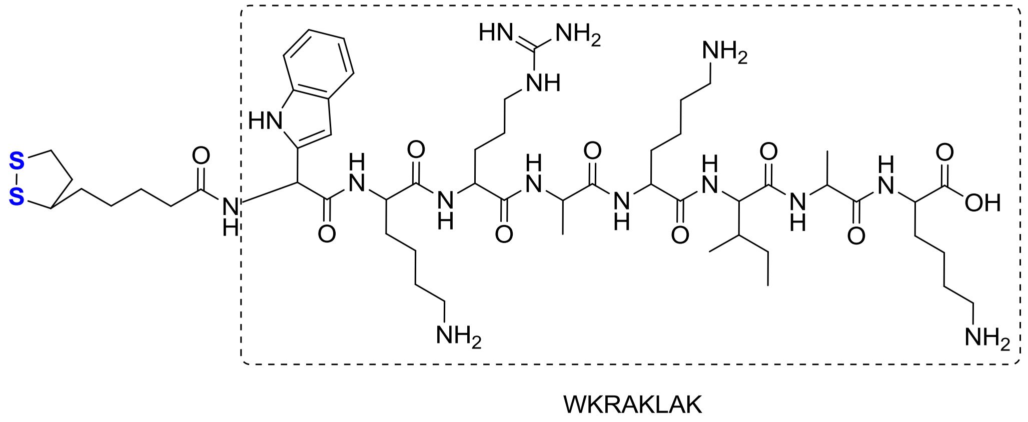

Figure S1. Primary structure of synthesized alpha-lipoic acid- peptide

Cell extract preparation. In the first step, MCF-7/T47D breast cancer cells were seeded at 8×10^4 cells per well in 24-well plates. Cells were treated with P-AuNPs at 37 °C in fresh media for 24h. Cells were then trypsinized, centrifuged at 12000 rpm and 4°C. To prepare the cell lysates, the pellet was resuspended in 60 μl of cold hypotonic buffer (containing 10 mM HEPES–KOH pH 7.5, 1.0 mM Na-EDTA, 10 mM KCl, 1.5 mM $MgCl_2$, 1.0 mM PMSF and 68 mM sucrose) and left on ice for 10min, followed by mixing for 30 sec by vortex. The insoluble materials were separated through centrifugation at 4°C and 15000 RCF for 30min. The supernatant of the cell lysates was stored at -80 °C until further analysis. The concentration of total protein in the cell lysates was determined using the Bradford protein assay.

Original Peptide

Peptide Sequence	Mutation Position	SVM score	Prediction	Hydrophilicity	Charge	pI	Mol wt
KLAKLAK	No	0.67	Anticp	0.63	3.00	10.31	771.11
KAAKLAK	2	0.66	Anticp	0.81	3.00	10.31	729.02
KCAKLAK	2	0.71	Anticp	0.74	3.00	9.81	761.08
KDAKLAK	2	0.70	Anticp	1.31	2.00	9.72	773.03
KEAKLAK	2	0.81	Anticp	1.31	2.00	9.72	787.06
KFAKLAK	2	0.47	Non-Anticp	0.53	3.00	10.31	805.12
KGAKLAK	2	0.75	Anticp	0.89	3.00	10.31	715.00
KHAKLAK	2	0.66	Anticp	0.81	3.50	10.31	795.09
KIAKLAK	2	0.43	Non-Anticp	0.63	3.00	10.31	771.11
KKAKLAK	2	0.70	Anticp	1.31	4.00	10.49	786.12
KMAKLAK	2	0.67	Anticp	0.70	3.00	10.31	789.14
KNAKLAK	2	0.60	Non-Anticp	0.91	3.00	10.31	772.05
KPAKLAK	2	0.70	Anticp	0.89	3.00	10.31	755.06
KQAKLAK	2	0.68	Anticp	0.91	3.00	10.31	786.08
KRAKLAK	2	0.83	Anticp	1.31	4.00	11.27	814.13
KSAKLAK	2	0.75	Anticp	0.93	3.00	10.31	745.02
KTAKLAK	2	0.74	Anticp	0.83	3.00	10.31	759.05
KVAKLAK	2	0.50	Non-Anticp	0.67	3.00	10.31	757.08

Modified peptide

Peptide Sequence	Mutation Position	SVM score	Prediction	Hydrophilicity	Charge	pI	Mol wt
WKRAKLAK	No	0.64	Anticp	0.72	4.00	11.27	1000.36

Table S1: Anticancer and other properties of the scanned sequence of KLAKLAK peptide predicted by AntiCP server

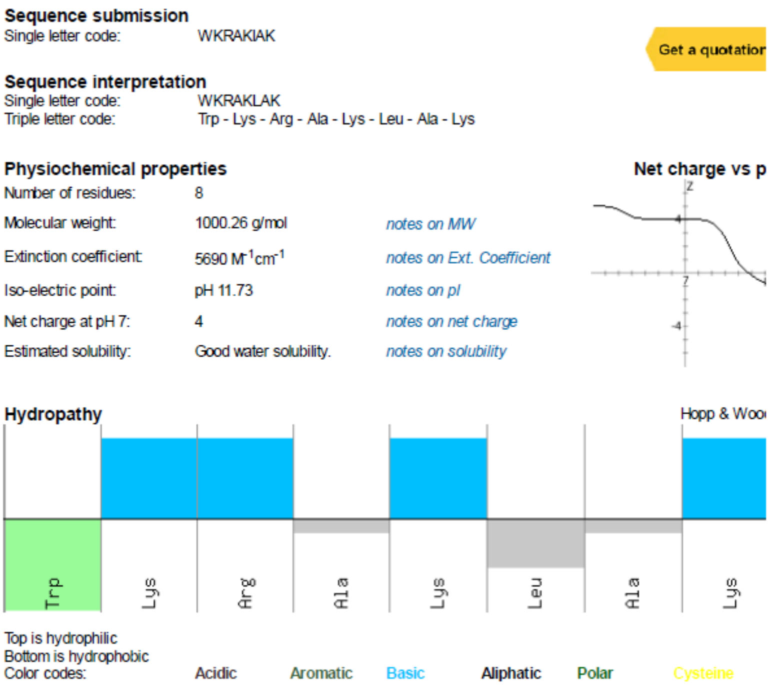

Figure S2: Properties of the synthesized peptide, calculated from innovagen peptide calculator (pepcalc.com).

.

HPLC system			
**Wavelength**	220nm		
**Mobile phase**	Solution A: (ACN:H2O)(70:30)		
	Solution B: Phosphate Buffer		
**Flow**	15ml/min		
**Gradient Method**	***Time***	***A%***	***B%***
	0	20	80
	15	80	20
	25	80	20
	35	100	0
	45	100	0

Table S2. Semipreparative HPLC conditions for LA-peptide conjugate purification

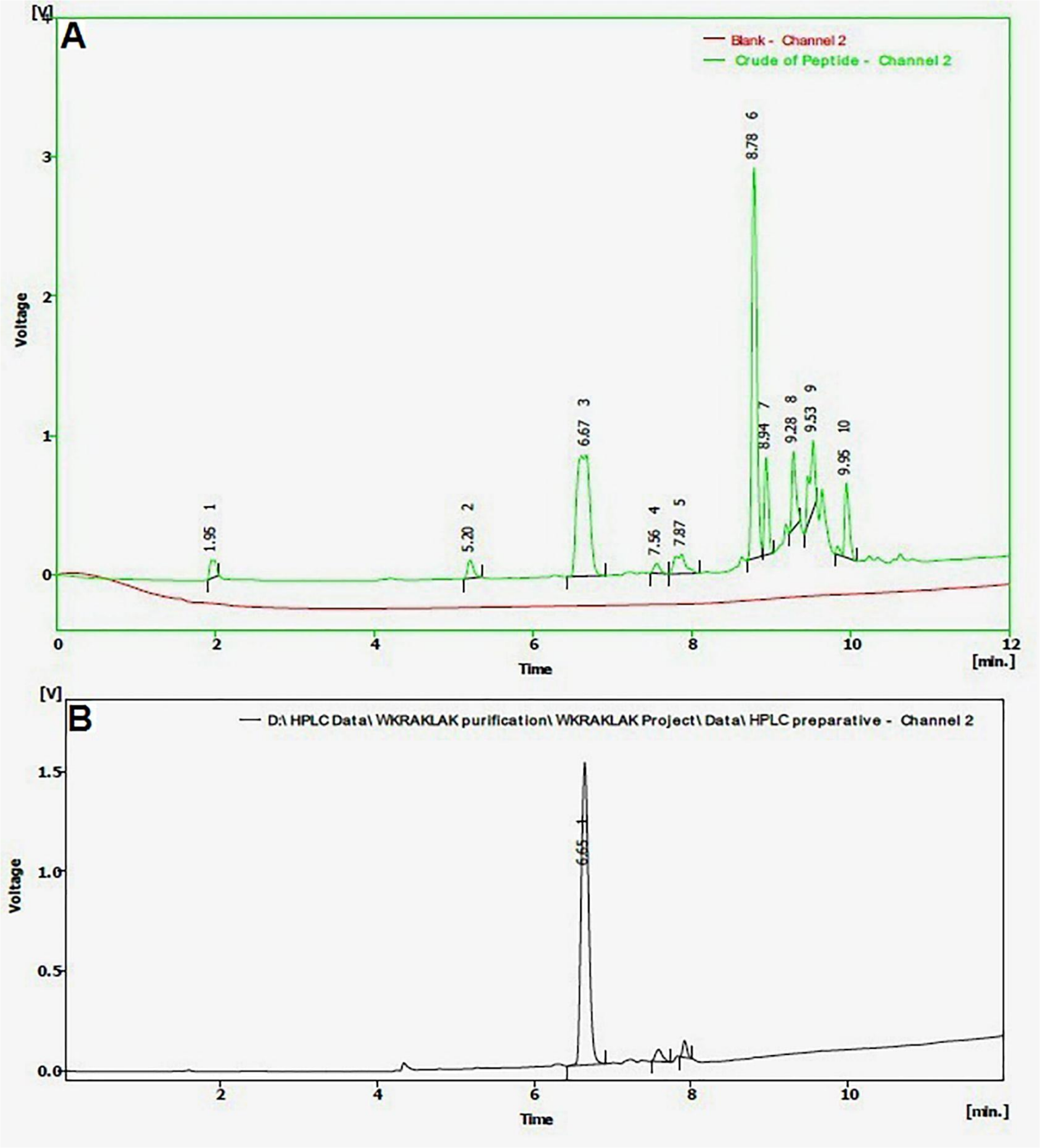

Figure S3: RP-HPLC chromatogram of (A) crude and (B) purified LA-peptide conjugate.

Gradient Table

	Time [min]	A [%]	B [%]	C [%]	D [%]	Flow [mL/min]
1	Initial	0.0	0.0	100.0	0.0	1.000
2	5.00	0.0	0.0	80.0	20.0	1.000
3	10.00	0.0	0.0	0.0	100.0	1.000
4	12.00	0.0	0.0	100.0	0.0	1.000
5						

Method Description: KRAKLAK Method

Column	C18
Mobile Phase	C) H2O D) ACN, 0.1%TFA
Flow Rate	1 ml/min
Pressure	900-1300 psi
Detection	UV 220nm
Temperature	30 C
Note	For hydrophilic peptide

☑ Enable Autostop

Run Time: 12 [min.]

☑ External Start/Stop

○ Start Only
◉ Start - Restart
○ Start - Stop

Table S3. Analytical RP-HPLC conditions for fractions of the peptide conjugate purified from semipreparative column.

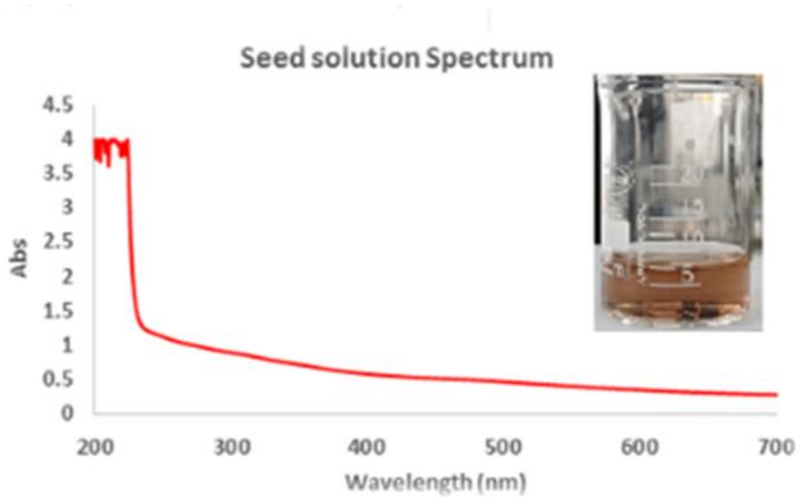

Figure S4. UV-vis spectra of Au seed solution (inset image shows the pale-brown-yellow color of seed solution).

LA-Peptide (nM)	0	50	85	135	170	210	150
λ for AuNS20 (nm)	524	526	529	530	531	531	531
λ for AuNS40 (nm)	533	535	536	536	536	537	537
λ for AuNR-LP720 (nm)	721	723	724	725	727	727	727
λ for AuNR-LP800 (nm)	800	803	805	811	813	813	813

Table S4. SPR shift of AuNPs in presence of different concentration of the LA-Peptide

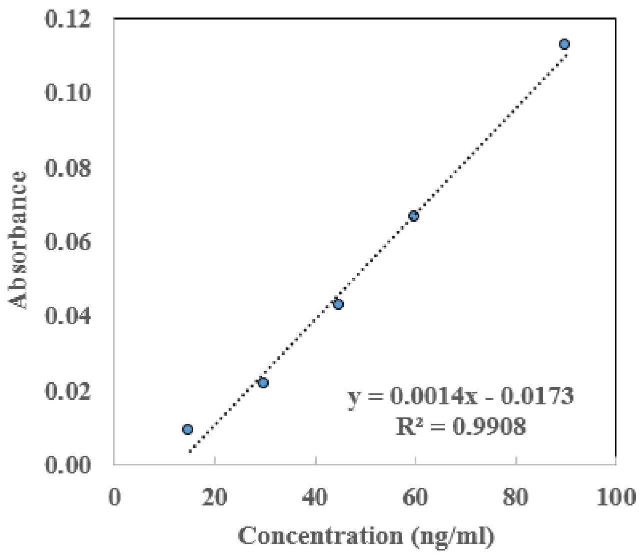

Figure S5. Calibration curve of gold standard by Atomic Absorption

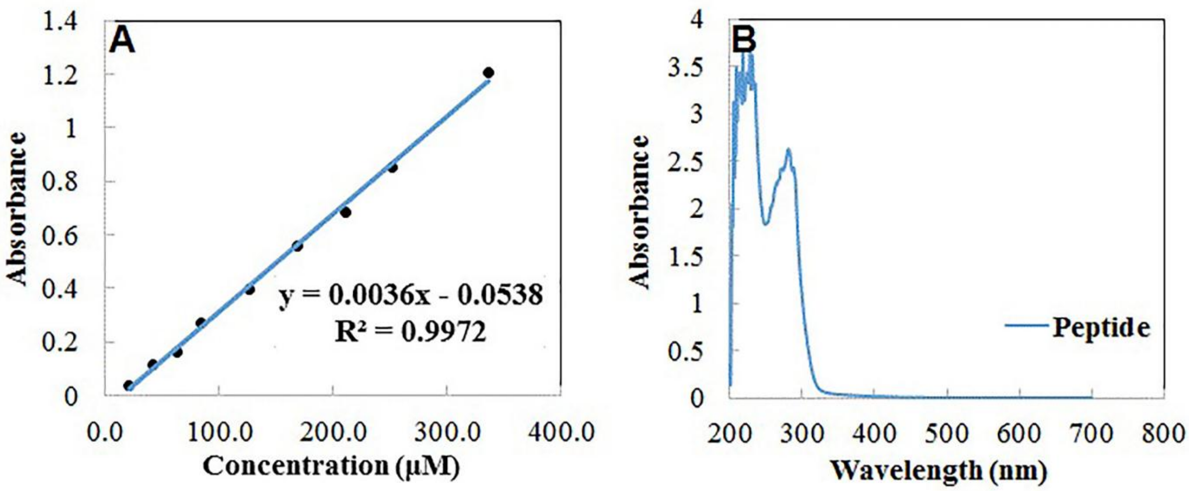

Figure S6. (A) Standard calibration curve for the LA-Peptide; (B) UV-Vis spectrum of the LA-Peptide.

Peptide-AuNPs		P-AuNS20	P-AuNS40	P-AuNR720	P-AuNR800	LA-Peptide
**Loading content for peptide (%)**		21	15	14	13	-
**IC_{50} for MCF-7 Cells**	µg/mL	8.75	13.13	21.25	41.90	258.7
	µM (Peptide content)	1.55	1.71	2.55	4.47	217.65
**IC_{50} for T47D Cells**	µg/mL	3.33	4.99	9.25	27.5	30% inhibition at IC_{50} for MCF-7 cells.
	µM (Peptide content)	0.59	0.65	1.11	2.93	

Table S5. Loading content of the LA-Peptide and calculated IC_{50} for P-AuNPs in terms of peptide content.

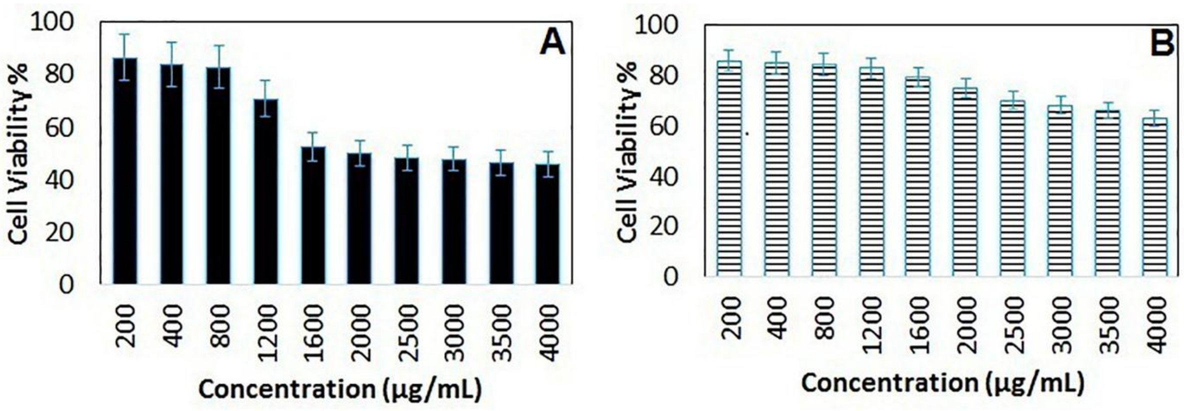

Figure S7. Cell viability of (A) MCF-7 and (B) T47D breast cancer cells exposed to the LA-Peptide.
